# Ventricular Tachycardia Caused by Mesothelial Cyst

**DOI:** 10.1016/s0972-6292(16)30588-5

**Published:** 2013-01-01

**Authors:** Ali Amirahmadi, Zahra Emkanjoo, Reza Mollazadeh, Kambiz Mozaffari

**Affiliations:** 1 Surgery department, Rajaee Cardiovascular Medical and Research Center (RCMRC), Tehran University of Medical Science (TUMS); 2 Electrophysiology Department, (RCMRC), TUMS; 3 Cardiology Department, Imam Khomeini Hospital, TUMS; 4 Pathologist (RCMRC), TUMS

**Keywords:** Pericardial cyst, Ventricular arrhythmia

## Abstract

A 27 year-old- lady was evaluated due to recurrent ventricular tachycardia. After performing echocardiography and cardiac MRI, she was found to have large pericardial cyst. Pathologic examination confirmed it as mesothelial pericardial cyst. Up to our knowledge it is the first presentation of simple pericardial cyst as ventricular a tachycardia.

A 27 year-old lady was evaluated due to repeated episodes of ventricular tachycardia (Figure 1A). She was referred for implantation of intracardiac cardiovertor-defibrillator. Transthoracic echocardiography showed a large cystic structure adjacent to posterior interventricular groove (Figure 1B). Cardiac MRI showed an encapsulated cyst in the cardiac crux containing high signal fluid with evidence of fibrosis in the adjacent myocardium (Figure 2A). The patient underwent surgery that showed a 5 x 4 cm cyst in the pericardium with adhesion to the underlying myocardium (Figure 2B). Pathologic examination showed the cyst to be mesothelial pericardial cyst.

## Discussion

Mesothelial pericardial cysts are benign intrathoracic lesions, usually found incidentally on chest X- ray in an asymptomatic patient or during investigation for common complaints such as chest pain and dyspnea [1]. Cyst attributable arrhythmia are rare and usually supraventricular (AF and AFL) caused mainly by compression over the atria or pulmonary veins. Herein, we reported an unusual presentation in our patient with a history of palpitation and documented VT. We highlight that the localized fibrosis of neighboring ventricular myocardium and VT may be present in an otherwise asymptomatic patient with pericardial cyst.

Simple pericardial cyst usually manifests as a non-enhanced, well defined mass in the pericardium with low intensity on T1-weighted and high intensity on T2 weighted. Rarely, as was our case, due to high protein content of the cyst, high signal intensity is seen on T1-weighted images [2].

This article emphasizes on the role of multi-imaging modality for accurate diagnosis in rare diseases esp. with uncommon presentations.

## Figures and Tables

**Figure 1:**
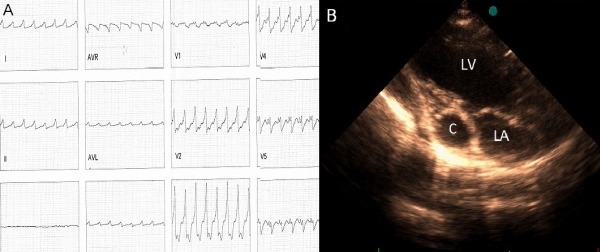
A-ECG shows wide QRS complex tachycardia confirmed during EP study as VT. B. Transthoracic echocardiography. Cyst (C) is obvious in atrioventricular groove relative to left atrium (LA) and left ventricle (LV).

**Figure 2:**
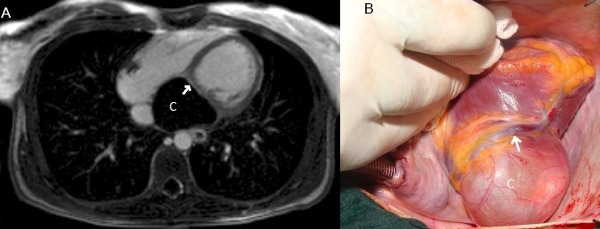
T1 (phase sequence inversion recovery) PSIR image shows the cyst (c) with late gadolinium enhancement in inferior wall of left and septal insertion site of right ventricle suggestive of fibrosis (Arrow). B. Intraoperative image: pericardial cyst (C) in close proximity to posterior descending artery (arrow).
